# Lats2-Underexpressing Bone Marrow-Derived Mesenchymal Stem Cells Ameliorate LPS-Induced Acute Lung Injury in Mice

**DOI:** 10.1155/2019/4851431

**Published:** 2019-10-21

**Authors:** Liang Dong, Lang Li

**Affiliations:** Department of Critical Care Medicine, Taizhou Central Hospital, Taizhou University Hospital, Taizhou, Zhejiang 318000, China

## Abstract

The pathophysiology of the acute lung injury (ALI) is characterized by the damage of alveolar epithelial cells, which can be repaired by exogenous bone marrow-derived mesenchymal stem cells (BMSCs). However, the migration and differentiation abilities of BMSCs are not sufficient for the purpose, and a new approach that could strengthen the repair effects of BMSCs in ALI still needs to be clarified. We have previously proved that *in vitro* large tumor suppressor kinase 2- (Lats2-) underexpressing BMSCs may enhance their tissue repair effects in ALI; thus, in the present study, we tried to explore whether Lats2-underexpressing BMSCs could rescue lipopolysaccharide- (LPS-) induced ALI *in vivo.* BMSCs from C57BL/6 mice transfected with Lats2-interfering lentivirus vector or lentivirus blank controls were transplanted intratracheally into LPS-induced ALI mice. The retention and differentiation of BMSCs in the lung were evaluated by *in vivo* imaging, immunofluorescence staining, and Western blotting. The lung edema and permeability were assessed by lung wet weight/body weight ratio (LWW/BW) and measurements of proteins in bronchoalveolar lavage fluid (BALF) using ELISA. Acute lung inflammation was measured by the cytokines in the lung homogenate and BALF using RT-qPCR and ELISA, respectively. Lung injury was evaluated by HE staining and lung injury scoring. Pulmonary fibrosis was evaluated by Picrosirius red staining, immunohistochemistry for *α*-SMA and TGF-*β*1, and hydroxyproline assay and RT-qPCR for Col1*α*1 and Col3*α*1. Lats2-mediated inhibition of the Hippo pathway increased the retention of BMSCs and their differentiation toward type II alveolar epithelial cells in the lung. Furthermore, Lats2-underexpressing BMSCs improved lung edema, permeability of the lung epithelium, and lung inflammation. Finally, Lats2-underexpressing BMSCs alleviated lung injury and early pulmonary fibrosis. Our studies suggest that underexpression of Lats2 could further enhance the repair effects of BMSCs against epithelial impair and the therapeutic potential of BMSCs in ALI mice.

## 1. Introduction

The pathophysiological hallmark of acute lung injury (ALI) is the disruption of alveolar epithelial cells, leading to increased permeability of blood–air barrier and noncardiogenic lung edema [1]. Currently, there are few effective pharmacological and supportive therapies for patients with ALI, and the mortality rate for moderate to severe ALI is 32%-45% [2]. Stem-cell-based repair of damaged alveolar epithelium will be a promising cure for patients with ALI [3]. Because of their properties of multipotency and immunoregulation, mesenchymal stem cells (MSCs) are ideal seed cells for cell-based therapy in ALI [4]. Recent animal and human studies reported that exogenous MSCs could migrate to ALI lung tissue and repair alveolar epithelial injury [5, 6]. However, the therapeutic effects remain limited due to the low engraftment and differentiation rates of MSCs in the lung tissue of ALI models [7, 8]. Therefore, clarifying the mechanisms that promote the differentiation, migration, and antioxidative activities of MSC may optimize the MSC-mediated therapeutic effects in ALI [9, 10].

The Hippo pathway, which depends on the accumulation of large tumor suppressor kinase 2 (Lats2) (NCBI Reference Sequence: NM_015771.2; http://www.ncbi.nlm.nih.gov/genbank), is one of the pivotal signaling pathways in the proliferation, differentiation, migration, and antiapoptotic activities of diverse cell types during organ size control, tissue homeostasis, and tumorigenesis [9]. Several recent studies have revealed that Lats2 and its downstream signaling have critical effects on the self-renewal, differentiation, proliferation, migration, and other biological functions of MSCs, which express a number of ligands, receptors, kinases, transcriptional regulators, and transcription factors of the Hippo pathway.

In our previous *in vitro* study, Lats2-underexpressing BMSCs derived from C57BL/6 mice were constructed successfully, and we found that Lats2-mediated inhibition of the Hippo pathway *in vitro* enhances the migration of bone marrow-derived MSCs (BMSCs) to injured lung tissue, promotes the differentiation of BMSCs into type II alveolar epithelial (ATII) cells, and confers resistance to H_2_O_2_-induced oxidative stress [11]. However, the effect of the Hippo pathway on the fate and therapeutic potential of BMSCs in ALI remains unclear *in vivo*. The microenvironment and regulatory mechanisms *in vivo* are more complicated and different from the particular and simple cultural conditions *in vitro*, and more evidence is needed to clarify the effect of the Hippo pathway on the protective role of BMSCs in the lungs of lipopolysaccharide- (LPS-) induced ALI mice. Therefore, the aim of our current study was to confirm the effect of Lats2-underexpressing BMSCs on the repair of injured alveolar epithelium and its potential therapeutic effect in LPS-induced ALI mice.

## 2. Materials and Methods

### 2.1. Transfection and Culture of BMSCs

BMSCs derived from C57BL/6 mice were obtained from Cyagen Biosciences, Inc. (Santa Clara, CA, USA). Lentivirus vector-mediated transfection of BMSCs was performed as previously described in our study [11]. After successful and stable transfection, BMSCs carrying enhanced green fluorescence protein (eGFP) and BMSCs carrying the Lats2-shRNA gene or the empty vector were cultured and passaged in a 1 : 1 mix of Dulbecco's modified Eagle's medium/nutrient mixture F-12 (DMEM/F12) culture medium (Thermo Fisher Scientific, Inc., Waltham, MA, USA) containing 10% fetal bovine serum (FBS) (Sigma-Aldrich, St Louis, MO, USA) and 1% streptomycin and penicillin at 37°C in a humidified atmosphere of 5% CO_2_. Passage 6 or 7 cells were used for subsequent experiments.

### 2.2. Preparation of Experimental Animals

Eighty male SPF C57BL/6 mice, aged 6-8 weeks and weighting 20-25 g, were obtained from the Research Animal Center of the Academy of Military Medical Sciences (Beijing, China). The mice were adapted to laboratory conditions for 3 days before experimentation. All animal use met the requirements of the Guide for the Care and Use of Laboratory Animals [12], and the experimental protocols were approved by the Institutional Animal Care and Use Committee at Taizhou University Hospital (Taizhou, China).

### 2.3. Murine Model of LPS-Induced ALI

The mice were first anesthetized by an intraperitoneal injection of pentobarbital (50 mg/kg), and ALI was produced by intratracheal instillation of a single dose of LPS (100 *μ*g) isolated from *Escherichia coli* 0111:B4 (Sigma-Aldrich, St Louis, MO, USA) in 50 *μ*l normal saline (NS). The mice were allowed to recover in an oxygenated chamber until fully awake.

### 2.4. Experimental Protocol

The mice were randomly allocated into four groups (*n* = 20 per group) as follows: normal control group, in which mice were initially injected intratracheally with 50 *μ*l of NS followed by 30 *μ*l of PBS after 4 h; ALI group, in which ALI was modeled by the intratracheal injection of 50 *μ*l of LPS followed by 30 *μ*l of PBS after 4 h; ALI+MSC-short hairpin RNA (sh) control group, in which mice with ALI were intratracheally transplanted with BMSCs transfected with empty lentivirus (5 × 10^4^ cells in 30 *μ*l PBS) 4 h after the production of ALI; ALI+MSC-shLats2 group, in which mice with ALI were intratracheally transplanted with BMSCs transfected with Lats2-interfering lentivirus (5 × 10^4^ cells in 30 *μ*l PBS) 4 h after the production of ALI. Mice were sacrificed 24 h and 3, 7, and 14 days later, and samples were collected for subsequent experiments.

### 2.5. Lung Histopathological Assessment

The whole lung was removed and inflated via the trachea with 4% paraformaldehyde in PBS at room temperature under a pressure of 20 cmH_2_O to fix the tissue. Lung tissue from the right upper lobe was dehydrated in graded concentrations of ethanol and then embedded in paraffin and transversely cut into 5-*μ*m-thick sections. Sections were stained with a HE Stain Kit (Abcam, Cambridge, MA, USA) according to the manufacturer's instructions. According to a previously published scoring method [13], the severity of lung injury was quantified blindly by a pathologist on the basis of images of ten randomly selected fields at ×200 magnification for each section, and the mean sum of each field score was calculated.

### 2.6. Pulmonary Fibrosis Assessment

Lung tissue from the right lower lobe was fixed in 4% paraformaldehyde at 4°C for 24 h, embedded in paraffin, and transversely cut into 4-*μ*m-thick sections. Sections were stained with a Picrosirius Red Stain Kit (Abcam, Cambridge, MA, USA) according to the manufacturer's instructions. Five randomly selected microscopic fields from 3 nonadjacent sections were captured by a light microscope connected to a digital camera. Quantitative assessment of lung collagen with Picrosirius red staining was performed using Image-Pro Plus 5.1 (Media Cybernetics, Rockville, MD, USA), and the results were expressed as a percentage of the total area of the image analyzed as previously described [14].

### 2.7. Hydroxyproline Assay

Hydroxyproline content of lung homogenates, which reflects the amount of collagen in the lung tissue, was measured by a Hydroxyproline Assay Kit (Abcam, Cambridge, MA, USA) according to the manufacturer's protocol. The absorbance of samples at 550 nm was read by a microplate reader (Bio-Rad, Hercules, CA, USA). The results were expressed as microgram per milligram lung weight.

### 2.8. Immunohistochemistry for Alpha-Smooth Muscle Actin (*α*-SMA) and Transforming Growth Factor-Beta1 (TGF-*β*1)

With the standard protocol being followed prior to HE staining described above, the sections were subjected to antigen retrieval and blocking as previously described [15]. For the immunohistochemical analysis, the sections were initially incubated with mouse anti-*α*-SMA antibody (Abcam, Cambridge, MA, USA) or mouse anti-TGF-*β*1 antibody (Thermo Fisher Scientific, Inc., Waltham, MA, USA) at 4°C overnight and were then incubated with HRP-conjugated goat antimouse secondary antibody (Abcam, Cambridge, MA, USA) at 37°C for 30 min. Positive staining was detected with HRP-conjugated streptavidin, visualized with 3,3′-diaminobenzidine, and counterstained with hematoxylin. Finally, the sections were mounted and cover-slipped, and images of five representative fields at ×400 magnification were captured by the Leica QWin Plus v3 software (Leica Microsystems, Cambridge, UK). The sizes of the study areas and the integrated optical density (IOD) of the positive stains were measured using Image-Pro Plus 5.1 (Media Cybernetics, Rockville, MD, USA). The expression levels of the proteins were expressed as the mean optical density (MOD; MOD = IOD per unit of study area).

### 2.9. Determination of Cytokines and Collagen Gene Expression by Real-Time Quantitative PCR (RT-qPCR)

Lung samples were homogenized in TRIzol Reagent (Thermo Fisher Scientific, Inc., Waltham, MA, USA), and total RNA was extracted according to manufacturer's instructions. Total RNA (2 *μ*g) was added to the reverse transcription reaction to synthesize cDNA. An ABI Prism 7900HT real-time quantitative PCR system (Applied Biosystems, Foster City, CA, USA) was used for product amplification. Cycling parameters were denaturation at 95°C for 15 seconds and annealing at 60°C for 1 minute, for 40 cycles. Reactions were performed in triplicate for each sample. The 2^−*ΔΔ*CT^ method was used to calculate the relative expression of mRNA, and GAPDH was used as the internal reference gene. The following primers were used: interleukin- (IL-) 1*β* forward, 5′-GTGCAAGTGTCTGAAGCAGC-3′, and reverse, 5′-CAAAGGTTTGGAAGCAGCCC-3′; IL-6 forward, 5′-GGAGTCACAGAAGGAGTGGC-3′, and reverse, 5′-CGCACTAGGTTTGCCGAGTA-3′; IL-4 forward, 5′-ACAGGAGAAGGGACGCCAT-3′, and reverse, 5′-GAAGCCCTACAGACGAGCTCA-3′; IL-10 forward, 5′-GGTTGCCAAGCCTTATCGGA-3′, and reverse, 5′-ACCTGCTCCACTGCCTTGCT-3′; Collagen Type I Alpha 1 (Col1*α*1) forward, 5′-ACATGTTCACGTTTGTGGACC-3′, and reverse, 5′-TAGGCCATTGTGTATGCAGC-3′; Collagen Type III Alpha 1 (Col3*α*1) forward, 5′-CCTTCTACACCTGCTCCT-3′, and reverse, 5′-CTTCCTGACTCTCCATCCT-3′; GAPDH forward, 5′-TCTCCTGCGACTTCAACA-3′, and reverse, 5′-TGTAGCCGTATTCATTGTCA-3′.

### 2.10. Labeling and Tracking of BMSCs

The cultured MSC-shcontrol and MSC-shLats2 cells were labeled with CellVue NIR815 dye (Thermo Fisher Scientific, Inc., Waltham, MA, USA) according to the manufacturer's instructions. NIR815-labeled cells (5 × 10^5^ cells) were directly transferred to the trachea of the ALI+MSC-shcontrol and ALI+MSC-shLats2 groups. A Maestro II *in vivo* imaging system (excitation = 786 nm, emission = 814 nm, exposition time 4,000 ms, PerkinElmer Inc., Waltham, MA, USA) was used to capture the *ex vivo* lung images from three mice per group at 3, 7, and 14 days after cell transfer to monitor the retention of BMSCs in the lungs. The autofluorescence spectra were then unmixed based on their spectral patterns using Maestro™ 2.2 software (PerkinElmer Inc., Waltham, MA, USA). The fluorescence intensity of the lungs was measured by placing regions of interest (ROIs) on the lung, and the average signals were normalized to the exposure time and the ROI area (scaled counts/s).

### 2.11. Fluorescence Microscopy

Immunofluorescent staining for the detection of retention and differentiation of transferred BMSCs *in vivo* was conducted as previously described [16]. Briefly, lung tissue from the left upper lobes was fixed in 4% paraformaldehyde at 4°C for 24 h, embedded in optimal cutting temperature (OCT) compound (Agar Scientific, Stansted, Essex, UK), and transversely cut into 5-*μ*m-thick sections. Nuclei were stained with 4′,6-diamidino-2-phenylindole (DAPI) (Thermo Fisher Scientific, Inc., Waltham, MA, USA), and eGFP fluorescence was detected using a fluorescence microscope (Olympus Corporation, Tokyo, Japan). The retention or differentiation of transferred BMSCs was quantified based on the count of GFP-positive BMSCs or the ratio of the count of SPC-positive to the count of GFP-positive BMSCs in randomly selected high-power fields (400x) for each slide [17].

### 2.12. Western Blot Analysis

Total protein lysates were prepared using total protein extraction kit (EMD Millipore, Bedford, MA, USA) according to the manufacturer's instructions. The protein was separated by SDS-PAGE and electrotransferred to PVDF membranes (EMD Millipore, Bedford, MA, USA), which were blocked with Tris-buffered saline containing 0.1% Tween-20 and 5% skim milk powder at 25°C for 1 h and then incubated at 4°C overnight with primary antibodies against SPC and occludin (Santa Cruz Biotechnology, Inc., Santa Cruz, CA, USA). Immunoreactive bands for SPC and occludin protein were detected with enhanced chemiluminescence reagent (Thermo Fisher Scientific, Inc., Waltham, MA, USA) and imaged with X-ray film.

### 2.13. Measurement of Cytokines and Proteins in Bronchoalveolar Lavage Fluid (BALF)

BALF was collected by flushing 1 ml ice-cold PBS back and forth three times through a tracheal cannula and then centrifuged at 1,000 g at 4°C. Mouse cytokine and protein enzyme-linked immunosorbent assay (ELISA) kits (Abcam, Cambridge, MA, USA) were used to measure the concentrations of interleukin- (IL-) 1*β*, IL-6, IL-4, IL-10, total protein (TP), and albumin (ALB) in the supernatant according to the manufacturer's instructions.

### 2.14. Evaluation of Lung Edema

Lung edema was measured based on the ratio of lung wet weight to body weight (LWW/BW). Briefly, the intact lung was harvested and trimmed to remove extrapulmonary tissues, and the LWW/BW was calculated based on the recorded lung wet weight and body weight [18]. The results were expressed in milligram per gram.

### 2.15. Statistical Analysis

Statistical analyses were performed using SPSS 22.0 software package (IBM, Armonk, NY, USA). All data are presented as the mean ± standard deviation, and a comparison among multiple groups was performed using a one-way analysis of variance followed by Bonferroni's *post-hoc* test. Values of *P* < 0.05 were considered statistically significant.

## 3. Results

### 3.1. Underexpression of Lats2 Increases the Retention of BMSCs in ALI Lung Tissue

**Figure 1:**
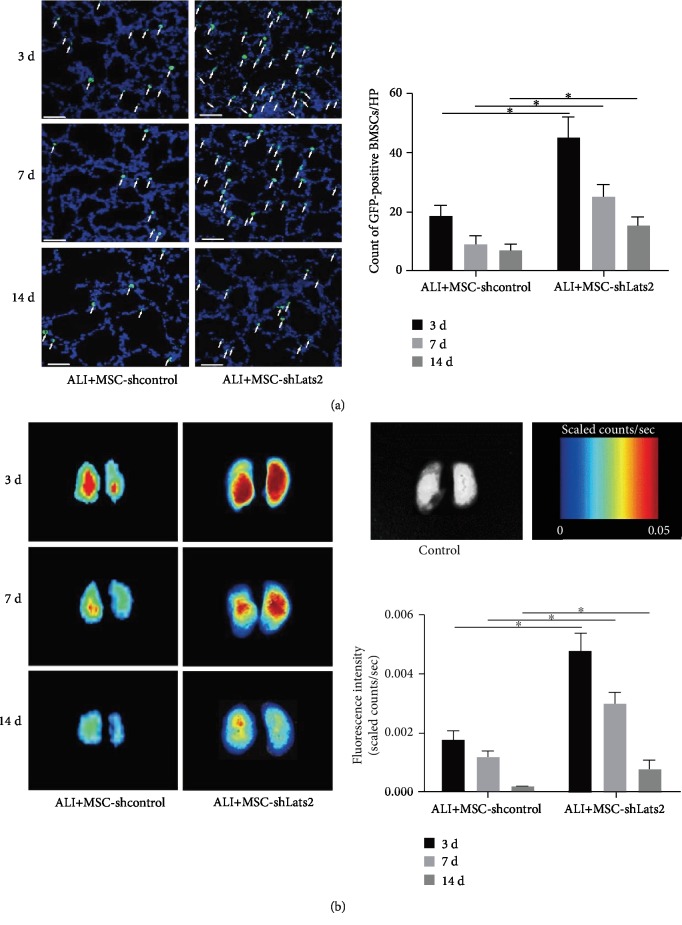
Underexpression of Lats2 increases the retention of BMSCs in ALI lung tissue. (a) Immunofluorescence staining images of lungs in the MSC-shcontrol and MSC-shLats2 groups are presented from six mouse lungs obtained 3, 7, and 14 days after LPS challenge. The nuclei were stained with DAPI (blue), and the engrafted BMSCs in the lung tissue are shown as GFP-positive (green; magnification, ×400; scale bar = 20 *μ*m; white arrows, GFP-positive cells). The count of GFP-positive BMSCs in randomly selected high-power fields is presented as the mean ± standard deviation (*n* = 6). (b) *Ex vivo* NIR imaging of lungs in the MSC-shcontrol and MSC-shLats2 groups are shown from six mouse lungs obtained 3, 7, and 14 days after LPS challenge. ^∗^*P* < 0.05.

Immunofluorescence staining and *ex vivo* near infrared region (NIR) imaging were performed on the lungs from ALI+MSC-shcontrol and ALI+MSC-shLats2 mice at 3, 7, and 14 days after LPS challenge to track the intrapulmonary BMSCs. Fluorescence microscopy revealed that the count of GFP-positive BMSCs in the ALI+MSC-shLats2 group was greater than that in the ALI+MSC-shcontrol group at 3, 7, and 14 days after BMSC administration (*P* < 0.05) (Figure 1(a)), and for each group, the count of GFP-positive BMSCs gradually decreased over time. Color-coded fluorescence imaging for detection of the BMSCs in lung tissue also observed similar results (Figure 1(b)).

### 3.2. Underexpression of Lats2 Promotes the Differentiation of BMSCs into ATII Cells

**Figure 2:**
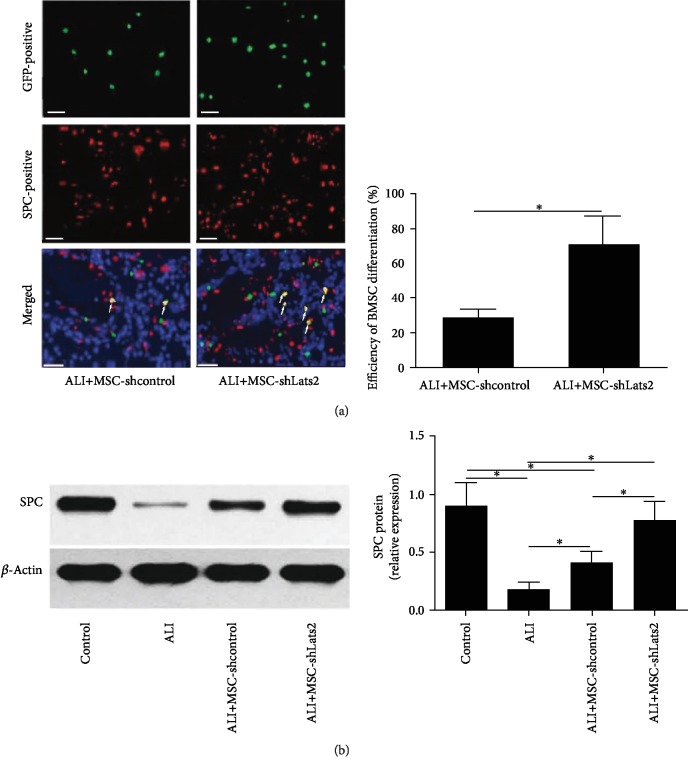
Underexpression of Lats2 promotes the differentiation of BMSCs into ATII cells. (a) Immunofluorescence staining images of lungs in the MSC-shcontrol and MSC-shLats2 groups are presented from six mouse lungs obtained 14 days after LPS challenge. Engrafted BMSCs, ATII cells, or BMSCs that differentiated into ATII cells in the lung are shown as GFP-positive (green), SPC-positive (red), or double-positive (yellow) under fluorescence microscopy, respectively. The nuclei were stained with DAPI (blue; magnification, ×400; scale bar = 20 *μ*m). The ratio of the count of SPC-positive to the count of GFP-positive BMSCs in randomly selected high-power fields is presented as the mean ± standard deviation (*n* = 6). (b) The protein expression of SPC in the lung tissue was measured using Western blot analysis 14 days after LPS challenge. *β*-Actin was used as the internal reference, and the results are presented as the mean ± standard deviation (*n* = 6). ^∗^*P* < 0.05.

Immunofluorescence staining and Western blot detection of the expression of SPC, a specific ATII cell marker, in the engrafted BMSCs were performed at 14 days after LPS challenge to evaluate the efficacy of BMSC differentiation on ATII cells. Immunofluorescence staining indicated that colocalization of BMSCs (green) and SPC (red) in the lung tissue (yellow) could be seen in both the ALI+MSC-shcontrol and ALI+MSC-shLats2 groups; however, underexpression of Lats2 led to a higher efficiency of the differentiation of BMSCs into AT II cells in the ALI+MSC-shLats2 group than in the ALI+MSC-shcontrol group (*P* < 0.05) (Figure 2(a)). Moreover, Western blot analysis revealed that SPC protein was upregulated in the ALI+MSC-shcontrol compared with the ALI group (*P* < 0.05) (Figure 2(b)), and SPC protein was further upregulated in the ALI+MSC-shLats2 group than in the ALI+MSC-shcontrol group (*P* < 0.05) (Figure 2(b)).

### 3.3. Lats2-Underexpressing BMSCs Improve Permeability of the Lung Epithelium and Lung Edema

**Figure 3:**
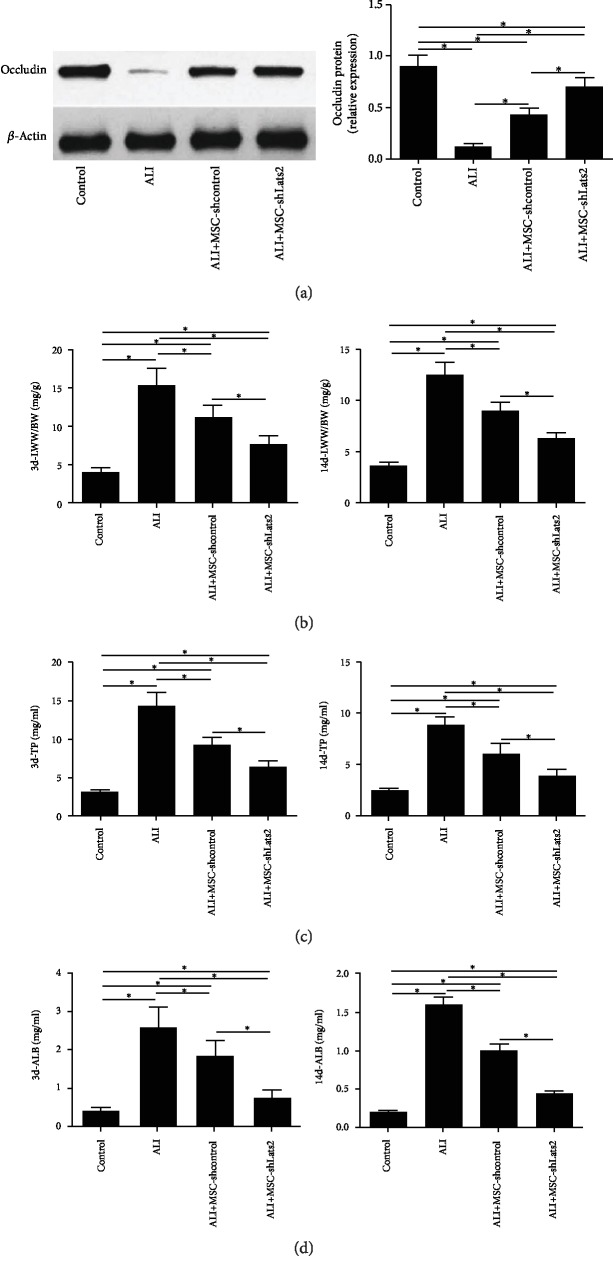
Lats2-underexpressing BMSCs improve lung edema and permeability of lung epithelium. (a) Protein expression of occludin in the lung tissue was measured using Western blot analysis at 14 days after LPS exposure. *β*-Actin was used as the internal reference. (b) Lung edema was measured by the LWW/BW at 3 and 14 days after LPS challenge. (c) TP in the BALF was measured using ELISA at 3 and 14 days after LPS challenge. (d) ALB in the BALF was measured using ELISA at 3 and 14 days after LPS challenge. The results are presented as the mean ± standard deviation of values obtained from six mice per group at each time point. ^∗^*P* < 0.05.

Expression of occludin protein was measured using Western blot to evaluate the tight junctions of the lung epithelium at 14 days after LPS challenge [18]. Occludin protein was significantly upregulated in the MSC-shcontrol group compared with the ALI group (*P* < 0.05) (Figure 3(a)), and occludin protein was further upregulated in the ALI+MSC-shLats2 group than in the ALI+MSC-shcontrol group (*P* < 0.05) (Figure 3(a)).

LWW/BW was measured to evaluate lung edema. LWW/BW was significantly reduced in the MSC-shcontrol group compared with the ALI group at 3 and 14 days after LPS challenge (*P* < 0.05) (Figure 3(b)), and LWW/BW was further reduced in the ALI+MSC-shLats2 group than in the ALI+MSC-shcontrol group at 3 and 14 days (*P* < 0.05) (Figure 3(b)).

The TP and ALB concentrations in the BALF were measured using ELISA to evaluate the epithelial permeability of the lung epithelium at 3 and 14 days after LPS challenge [19]. TP and ALB in the BALF were significantly higher in ALI group than in the control group at 3 and 14 days (*P* < 0.05) (Figure 3(c)). However, TP and ALB in the BALF were significantly reduced in the MSC-shcontrol group compared with the ALI group at 3 and 14 days (*P* < 0.05) (Figures 3(c) and 3(d)), and TP and ALB in the BALF were further reduced in the ALI+MSC-shLats2 group than in the ALI+MSC-shcontrol group at 3 and 14 days (*P* < 0.05) (Figures 3(c) and 3(d)).

### 3.4. Lats2-Underexpressing BMSCs Improve Acute Lung Inflammation

**Figure 4:**
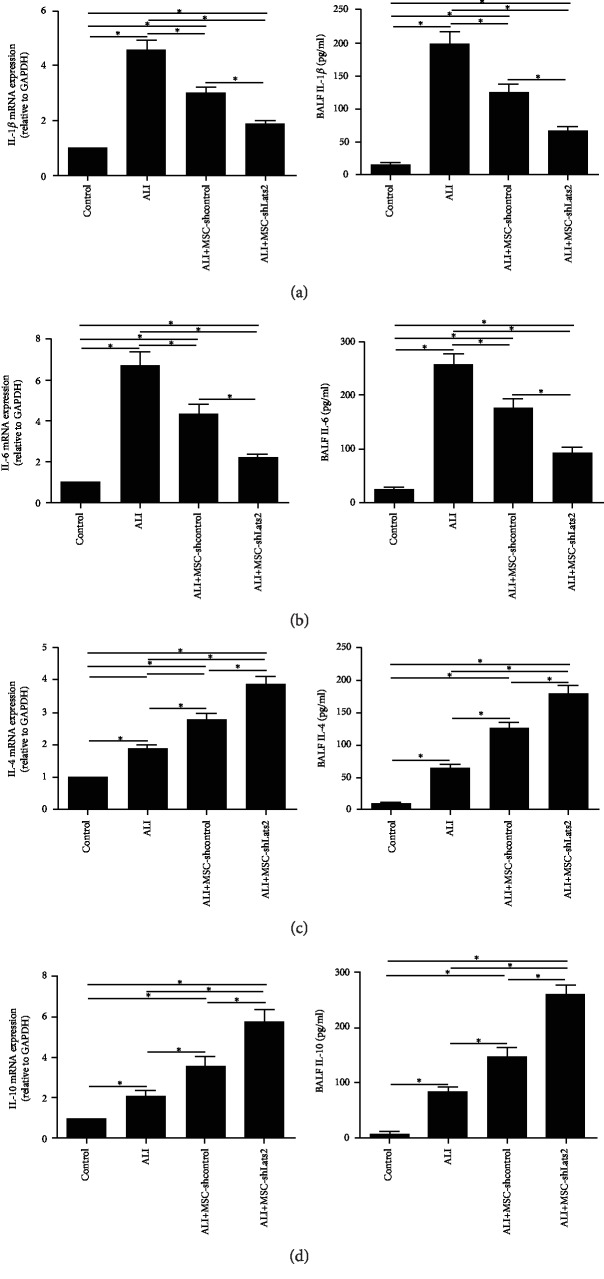
Lats2-underexpressing BMSCs improve acute lung inflammation. The mRNA expressions of proinflammatory cytokines (a) IL-1*β* and (b) IL-6 and anti-inflammatory cytokines (c) IL-4 and (d) IL-10 in the lung homogenate were measured using RT-qPCR at 24 h after LPS challenge. GAPDH was used as the internal reference gene (*n* = 3). The concentrations of (a) IL-1*β*, (b) IL-6, (c) IL-4, and (d) IL-10 in the BALF were measured using ELISA at 3 days after LPS challenge (*n* = 6). Data are presented as the mean ± standard deviation. ^∗^*P* < 0.05.

The mRNA expressions of the proinflammatory cytokines IL-1*β* and IL-6 and the anti-inflammatory cytokines IL-4 and IL-10 in the lung homogenate at 24 h after LPS challenge and their protein levels in the BALF at 3 days after LPS challenge were measured by RT-qPCR and ELISA to evaluate acute lung inflammation. The mRNA and protein levels of all four cytokines were significantly higher in the ALI group than in the control group (*P* < 0.05) (Figure 4). The mRNA and protein levels of IL-1*β* and IL-6 were significantly reduced in the MSC-shcontrol group compared with the ALI group (*P* < 0.05) (Figures 4(a) and 4(b)), and their levels were further reduced in the ALI+MSC-shLats2 group than in the ALI+MSC-shcontrol group (*P* < 0.05) (Figures 4(a) and 4(b)). However, the mRNA and protein levels of IL-4 and IL-10 were significantly increased in the MSC-shcontrol group compared with the ALI group (*P* < 0.05) (Figures 4(c) and 4(d)), and their levels were further increased in the ALI+MSC-shLats2 group than in the ALI+MSC-shcontrol group (*P* < 0.05) (Figures 4(c) and 4(d)).

### 3.5. Lats2-Underexpressing BMSCs Alleviate Pathological Injuries in ALI Lung Tissue

**Figure 5:**
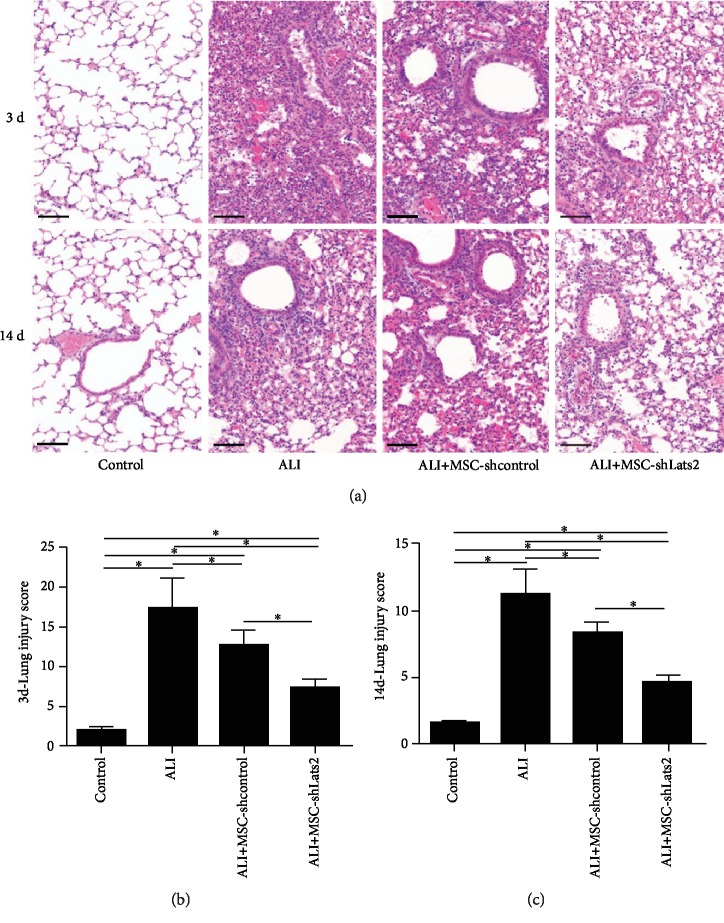
Lats2-underexpressing BMSCs alleviate pathological injuries in ALI lung tissue. (a) Histopathological analysis of lung tissues was performed at 3 and 14 days after LPS challenge. (HE staining; magnification, ×200; scale bar = 100 *μ*m). (b) Lung injury score at 3 days after LPS challenge. (c) Lung injury score at 14 days after LPS challenge. Lung injury score is expressed as arbitrary unit. Data are presented as the mean ± standard deviation (*n* = 3). ^∗^*P* < 0.05.

HE staining and lung injury score were used to evaluate lung injuries at 3 and 14 days after LPS challenge. Alveolar wall thickening, diffused infiltration of inflammatory cells, hemorrhage, intra-alveolar exudates, and edema were found in the lung tissue of ALI group at 3 and 14 days (Figure 5(a)), and the lung injury score was also higher than that in the control group (*P* < 0.05) (Figures 5(b) and 5(c)). However, these pathological changes and the lung injury score were significantly reduced in the MSC-shcontrol group compared with the ALI group at 3 and 14 days (*P* < 0.05) (Figures 5(b) and 5(c)), and these pathological changes and the lung injury score were further reduced in the ALI+MSC-shLats2 group than in the ALI+MSC-shcontrol group at 3 and 14 days (*P* < 0.05) (Figures 5(b) and 5(c)).

### 3.6. Lats2-Underexpressing BMSCs Inhibit Early Pulmonary Fibrosis

**Figure 6:**
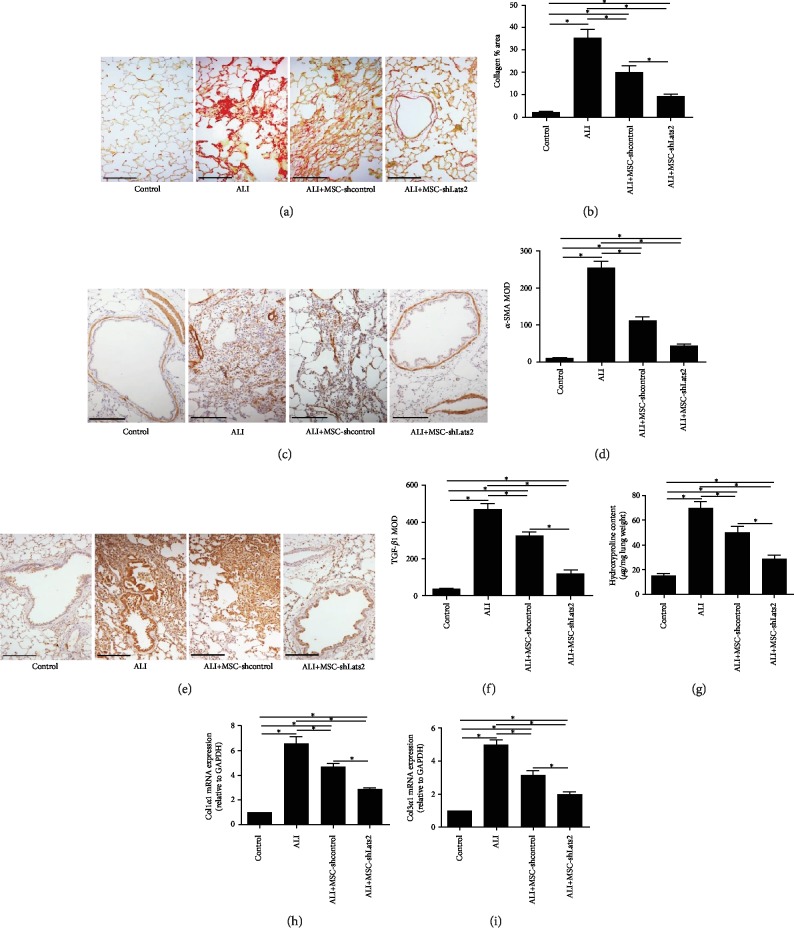
Lats2-underexpressing BMSCs inhibit early pulmonary fibrosis. (a) Picrosirius red staining of collagen fibers (red staining) in the lung tissues at 14 days after LPS challenge. (Picrosirius red staining; magnification, ×200; scale bar = 50 *μ*m). (b) Quantification of collagen area in Picrosirius red stained-lung sections at 14 days after LPS challenge. (c) Immunohistochemistry for *α*-SMA (dark brown staining) in the lung tissues at 14 days after LPS challenge (magnification, ×400; scale bar = 200 *μ*m). (d) Quantification of mean optical density (MOD) for *α*-SMA in the lung tissues at 14 days after LPS challenge. (e) Immunohistochemistry for TGF-*β*1 (dark brown staining) in the lung tissues at 14 days after LPS challenge (magnification, ×400; scale bar = 200 *μ*m). (f) Quantification of MOD for TGF-*β*1 in the lung tissues at 14 days after LPS challenge. (g) Hydroxyproline content of lung homogenates at 14 days after LPS challenge. (h) The mRNA expression of Collagen Type I Alpha 1 (Col1*α*1) in the lung tissue at 7 days after LPS challenge. GAPDH was used as the internal reference gene. (i) The mRNA expression of Collagen Type III Alpha 1 (Col3*α*1) in the lung tissue at 7 days after LPS challenge. GAPDH was used as the internal reference gene. Data are presented as the mean ± standard deviation (*n* = 3). ^∗^*P* < 0.05.

Picrosirius red staining, immunohistochemistry, and hydroxyproline assay at 14 days after LPS challenge and mRNA expressions of Col1*α*1 and Col3*α*1 at 7 days after LPS challenge were measured to evaluate early pulmonary fibrosis. Thickening of alveolar and bronchiolar walls, damage to lung structure, formation of fibrous bands or fibrous masses, and fibrous obliteration were found in the lung tissue of ALI group (Figure 6(a)). The area of fibrosis (Figure 6(b)), the MOD for *α*-SMA and TGF-*β*1 (Figures 6(c)–6(f)), hydroxyproline content (Figure 6(g)), and mRNA expressions of Col1*α*1 and Col3*α*1 (Figures 6(h) and 6(i)) were also higher than those in the control group (*P* < 0.05). However, these fibrotic changes (Figure 6(a)), the area of fibrosis (Figure 6(b)), the MOD for *α*-SMA and TGF-*β*1 (Figures 6(c)–6(f)), hydroxyproline content (Figure 6(g)), and mRNA expressions of Col1*α*1 and Col3*α*1 (Figures 6(h) and 6(i)) were significantly reduced in the MSC-shcontrol group compared with the ALI group (*P* < 0.05), and these parameters were further reduced in the ALI+MSC-shLats2 group than in the ALI+MSC-shcontrol group (*P* < 0.05) (Figure 6).

## 4. Discussion

MSC therapy may be a promising cure for ARDS because of its ability to migrate to and engraft injured lungs and to differentiate into alveolar epithelial cells *in vivo* [5, 6]. However, the beneficial effects of MSCs in ARDS are limited by the relatively low engraftment and differentiation rates of MSCs in the injured lungs [7, 8]. Our previous *in vitro* study indicated that Lats2-mediated inhibition of the Hippo pathway promoted the migration of MSCs to injured lung tissue, differentiation into ATII cells, and resistance to oxidative stress [11]. This indicated that modulation of the Hippo pathway may improve the therapeutic effects of MSCs in ARDS. In the present study, we confirmed the protective effect of the Hippo pathway in LPS-induced ALI using genetically modified Lats2-underexpressing BMSCs.

The Hippo pathway is an essential regulatory signaling in the development, proliferation, differentiation, and other physiological functions of cells and organs [20]. The Hippo pathway begins with the binding of the cell surface ligand dachsous homologs 1 and 2 to the cell surface receptor FAT tumor suppressor homolog 4 on a neighboring cell, which thus phosphorylates and activates the nuclear dbf2-related family kinase Lats2 [21]. Lats2 kinase phosphorylates the downstream transcriptional regulator Yes-associated protein (YAP), inhibiting the activity of the Hippo pathway [22]. Therefore, Lats2 was selected as the target gene for the regulation of the Hippo pathway. In our previous study, we have used lentiviral vectors to obtain BMSCs with highly efficient and stable transgenic underexpression of Lats2, which subsequently inhibited the Hippo pathway in BMSCs [11]. Moreover, we have also confirmed that Lats2-mediated inhibition of the Hippo pathway could enhance the proliferation, differentiation, and migration of BMSCs *in vitro* [11]. These findings suggested that Lats2-underexpressing BMSCs might optimize ARDS therapy, and we have confirmed the benefits of using Lats2-underexpressing BMSCs for ARDS as expected.

We found that underexpression of Lats2 increased the retention of BMSCs in ALI lung tissue. The retention of exogenous BMSCs in ALI lung tissue is a prerequisite for tissue repair [23]. In agreement with our findings, enhanced recruitment of exogenous MSCs to the lung tissue in ALI mice compared with normal control mice was observed [24], and the accelerating effect of inhibition of the Hippo pathway on the migration of MSCs has been demonstrated recently in other studies [24, 25]. Currently, the regulatory mechanisms of MSC migration remain unclear. It has been reported that TGF-*β*, IL-1*β*, tumor necrosis factor- (TNF-) *α*, stem cell-derived factor-1*α*, and C-X-C chemokine receptor type 4 may contribute to the engraftment of MSCs [26, 27]. The present study showed that the Hippo pathway also regulates the migration of BMSCs *in vivo*.

Underexpression of Lats2 also increased the differentiation of BMSCs into ATII cells. The differentiation of BMSCs into ATII cells represents the most important rationale for tissue repair [3]. This result is consistent with data reported in several previous studies, which observed the promoted differentiation of exogenous MSCs into ATII cells in the lung tissue in ALI mice compared with normal control mice [28], and the positive effect of inhibition of the Hippo pathway on the differentiation of MSCs into ATI cells has been demonstrated recently in other studies [29, 30]. However, several studies also found diverse roles of the Hippo pathway on the differentiation of MSCs, which depends on the disease, microenvironment, and treatment timing [31, 32]. The effects of the Hippo pathway on the differentiation of BMSCs in LPS-induced ALI mice are worth exploring in further studies.

In the present study, Lats2-underexpressing BMSCs improved occluding expression, LWW/BW, and level of BALF proteins in ALI lung tissue. Occludin is the hallmark of intact tight junction and provides most of the barrier function of tight junctions [33]. Disruption of the tight junction barrier and downregulation of occludin protein expression have been observed in ALI in rats [34]. Alveolar epithelial cells with compromised tight junctions show a significantly increased permeability, leading to lung edema and intra-alveolar protein exudate. Likewise, a previous study has found that MSC treatment significantly improved the repression of occludin expression, the lung LWW/BW, and the level of BALF proteins in experimental ALI [35].

We also observed that Lats2-underexpressing BMSCs improved acute lung inflammation in ALI lung tissue. One of the pathophysiologies of ALI is essentially dysregulated inflammation that manifested by imbalance between proinflammatory and anti-inflammatory response in the lung [36, 37]. Several previous studies indicated that MSCs could enhance the resolution of inflammation in adult ALI patients [16, 35], and sufficient evidence also showed the same modulatory effect: Proinflammatory cytokines such as interferon-*γ*, IL-1*β*, and IL-6 were reduced, while the anti-inflammatory cytokines IL-4 and IL-10 were increased [38].

We also found that the Lats2-underexpressing BMSCs alleviated pathological injuries in ALI lung tissue. The pathological alterations in the lung tissue are the key factors associated with ALI diagnosis, treatment effect, and patient prognosis [39]. Several mechanisms may contribute to the therapeutic effects of BMSCs for lung injuries, such as immunomodulation, antibacterial effect, enhanced alveolar fluid clearance, and improvement of lung permeability, which were also confirmed in the present study [40]. Our results are similar to those of several previous studies, which indicated that MSCs improved pathological injuries in ALI mice [16, 35].

Finally, we found that Lats2-underexpressing BMSCs inhibited early pulmonary fibrosis in ALI lung tissue. Early pulmonary fibrosis during the repair process may directly aggravate lung function and the long-term life quality of patients with ALI, and pulmonary fibrosis occurs as early as 3 days in ALI [41, 42]. Several mechanisms may contribute to the therapeutic effects of BMSCs for early pulmonary fibrosis, such as inhibition of TGF-*β*-smad2/3 pathway and reduced expression of matrix metalloproteinase- (MMP-) 2, 9, and 13 [43, 44]. Our results are similar to those of several previous studies, which indicated that MSCs reduced deposition of collagen fiber and improved early pulmonary fibrosis in ALI mice [16, 35]. Lats2-underexpressing BMSCs amplified this effect; however, the underlying mechanisms still need to be clarified.

Collectively, the present study demonstrated that inhibition of the Hippo pathway through the underexpression of Lats2 increased the retention of BMSCs in the lung and promoted the differentiation of BMSCs into AT II cells. Moreover, Lats2-underexpressing BMSCs further improved the lung epithelial permeability, ameliorated acute lung inflammation, and inhibited lung injuries and early pulmonary fibrosis in ALI mice, thus contributing to an optimized therapeutic effect of BMSCs in ALI; however, clinical trials are required.

## Data Availability

All data generated or analyzed during this study are included in this article.
